# Magneto-absorption spectra of hydrogen-like yellow exciton series in cuprous oxide: excitons in strong magnetic fields

**DOI:** 10.1038/s41598-018-25486-6

**Published:** 2018-05-18

**Authors:** Sergey Artyukhin, Dmitry Fishman, Clément Faugeras, Marek Potemski, Alexandre Revcolevschi, Maxim Mostovoy, Paul H. M. van Loosdrecht

**Affiliations:** 1 Istituto Italiano di Tecnologia, Genova, 16163 Italy; 2 Department of Chemistry, University of California, Irvine, CA 92697 USA; 3 Laboratoire National des Champs Magnétiques Intenses, CNRS-UGA-UPS-INSA-EMFL, 38042 Grenoble, France; 4 Institut de Chimie Moléculaire et des Materiaux d’Orsay - UMR 8182, Université de Paris Sud, Bâtiment 410, 91405 Orsay Cedex, France; 5 Zernike Institute for Advanced Materials, University of Groningen, 9747 AG Groningen, The Netherlands; 6 Physics Institute II, University of Cologne, Köln, 50937 Germany

## Abstract

We study the absorption spectra of the yellow excitons in Cu_2_O in high magnetic fields using polarization-resolved optical absorption measurements with a high frequency resolution. We show that the symmetry of the yellow exciton results in unusual selection rules for the optical absorption of polarized light and that the mixing of ortho- and para- excitons in magnetic field is important. The calculation of the energies of the yellow exciton series in strong and weak magnetic field limits suggests that a broad *n* = 2 line is comprized by two closely overlapping lines, gives a good fit to experimental data and allows to interpret the complex structure of excitonic levels.

## Introduction

Cuprous oxide Cu_2_O was the first material in which Wannier-Mott excitons^1^ – the electron-hole pairs bound by the Coulomb interaction – were observed. These states give rise to hydrogen-like series of absorption lines in the optical absorption spectrum of Cu_2_O at the photon energies described by the Rydberg formula, *E*_*n*_ = *E*_gap_ − Ry_X_/*n*^2^, where Ry_X_ = 98 meV is the excitonic Rydberg constant and *E*_gap_ = 2.17 eV is the optical gap. The only exception is the *n* = 1 exciton, which is dipole forbidden as the valence and conduction bands of this material have the same parity^2^. Due to the small size of the *n* = 1 exciton, its energy is strongly affected by exchange, central cell corrections and reduced screening of the Coulomb interaction^3^. The corrections to the binding energy of the higher levels, produced by these mechanisms, are negligible. The revival of interest in Cu_2_O was motivated by the search for the Bose-Einstein condensation of the exciton gas^4–8^ and rapid developments of ab-initio methods^9^, e.g. GW and Bethe-Salpeter calculations, for which Cu_2_O serves as an important benchmark system. These studies underscored the importance of a quantitative description of excitons in this material. Despite the recent progress^3,10^, a number of fundamental problems, surprisingly, remain unsolved. For example, there is a siginificant discrepancy between the effective masses of electrons and holes deduced from the optical measurements^11,12^ and the cyclotron resonance experiments^13^. In addition, a detailed interpretation of the magneto-optical spectra is still lacking^14–16^. In this paper we address these issues and resolve the discrepancy using high-resolution measurements of the low-energy magneto-optical absorption spectra of Cu_2_O combined with numerical calculations of the spectra in the intermediate magnetic field regime.

Optical absorption spectra measured in zero fields give information about the excitonic Rydberg constant and the reduced mass of the electron-hole pair. Further information can be obtained from the splitting of excitonic levels in applied electric or magnetic fields. The magnetoabsorption spectra of excitons in Cu_2_O were extensively studied over the past decades^11,14–18^. Recent measurements of higher levels *n* > 5 under magnetic fields up to 7 T compared the scaling of features in absorption spectra of Rydberg excitons in external fields to those of a hydrogen atom. While certain features, such as electric fields producing intersections (resonances) of levels with neighboring *n* scale in the same way in Rydberg excitons and hydrogen atoms, magnetic fields for similar resonances scale differently^19^. Because of the large dielectric constant and small effective masses of charge carriers in Cu_2_O, the exciton radius greatly exceeds that of a hydrogen atom, which amplifies the effects of external electric and magnetic fields on the exciton wave function. For example, the exciton ionization in Cu_2_O occurs in an electric field of *E* = 5 kV/cm^17^, whereas the characteristic field required to ionize the hydrogen atom is of the order of *E* ~ 1000 kV/cm. Pronounced and fairly complex Zeeman and Stark effects were first observed by Gross and Zakharchenya in magnetic fields up to 2.8 T^17^. Recently, excitons up to *n* = 25 were observed using single frequency dye laser with a line width of 5 neV for excitation^20^. These excitons are above 2 *μ*m in diameter. A reduction in excitonic absorption with increasing laser power due to Rydberg blockade, repulsion between large excitons, was demonstrated. Rydberg blockade could pave the way to single-photon logic devices^21^.

Due to the large radius of the Cu_2_O excitons, the strong magnetic field regime, in which the field-induced level splitting becomes comparable with the zero field level spacing, can be easily reached in laboratory conditions (for hydrogen atom such strong fields are only found in neutron stars). For a magnetic field of *H* = 30 T the cyclotron energy is comparable to the binding energy already for the *n* = 3 exciton. For higher levels, the strong-field regime is reached at even lower fields (*β*_*n*_ = *ħω*_*c*_/(2*Ry*_*X*_/*n*^2^) ≈ 0.05*n*^2^, where *ω*_*c*_ is the cyclotron frequency). However, the interpretation of experimentally measured spectra of exciton states with large *n* in applied magnetic fields is complicated by the large number of overlapping lines, which makes it difficult to extract exciton parameters from such spectra in a reliable way.

Zhilich *et al*.^12^ studied the oscillations of optical absorption well above the gap in magnetic fields up to 10 T. These oscillations originate from the transitions between the Landau levels of electrons and holes. The effective masses of electrons and holes were estimated to be *m*_*e*_ = 0.61*m*_0_, *m*_*h*_ = 0.84*m*_0_, where *m*_0_ is the bare electron mass. The accuracy of these results was limited by poor energy resolution and available magnetic fields. At lower energies, in the region of bound excitons, the absorption lines are much sharper and more suitable for extraction of exciton parameters. Sasaki and Kuwabara^14^ measured the magnetoabsorption spectrum in static magnetic fields up to 16 T. Kobayashi *et al*.^15^ studied the *n* = 2 and *n* = 3 exciton absorption in pulsed magnetic fields up to 150 T. Seyama *et al*.^16^ measured the spectra in static fields up to 25 T with better spectral resolution. However, the complexity of the spectra with large numbers of overlapping lines prevented the unambiguous assignment of excitonic levels. In addition, Coulomb interactions between the electron and hole were not taken into account in the analysis of the spectra.

The effective masses obtained from cyclotron resonance experiments^13^: 0.58*m*_0_ and 0.69*m*_0_ for light and heavy holes, respectively, and 0.99*m*_0_ for electrons are significantly different from the masses obtained in magnetooptical measurements (see Table 1). This disagreement was ascribed to polaronic effects^11,12^. More recently the value of 0.575*m*_0_ was derived from pulsed cyclotron resonance experiments^22^.

**Table 1:** Effective masses of electrons *m*_*e*_, holes *m*_*h*_, the dielectric constant *ε* and an exciton reduced mass *μ* obtained in previous experiments^12,13^ and in the present work.

Method	*m*_*e*_	*m*_*h*_	*μ*	*ε*	*g*_*c*_	*g*_*v*_
Optical^12^	0.61	0.84	0.35	7.1	2.0	0.28
Cyclotron resonance^13^	0.99	0.69	0.41	7.5		
Current work	1.0	0.7	0.4	7.5	2.1	−0.1

The masses are in the units of bare electron mass *m*_0_. The last two columns give the *g*-factors of the electrons and holes, respectively.

In the attempts to extract the exciton parameters from the spectra measured at high magnetic fields, the Coulomb energy of electron and hole was neglected; an approximation which is justified only for very large magnetic fields and large-*n* excitons. In works using low excitonic levels the magnetic field was treated perturbatively; an approximation only justified in a limited area of the spectra. Therefore, the most promising are the levels with *n* ≤ 5, falling into the intermediate field regime *β*_*n*_ ~ 1. There is no small parameter for a perturbative expansion, and numerical calculations are required to obtain the exciton spectrum.

We performed polarized, high spectral resolution optical absorption measurements in static magnetic fields up to 32 T and obtained the magnetic field dependence of the exciton energies for *n* = 2, 3 and 4 with high accuracy. The largest part of the spectrum lies in the intermediate-field regime, in which the interaction of electrons and holes with the magnetic field is comparable with Coulomb interaction, so that neither of these interactions can be treated perturbatively. We calculated the exciton energies in the intermediate regime numerically and extracted the effective masses and *g*-factors of the electron and hole by fitting the data. Not only do we get a good agreement between the theory and magnetoabsorption experiments, but the masses that we obtain coincide with those obtained from the cyclotron resonance experiments, thus resolving the long-standing contradiction.

This paper is organized as follows. In the Experimental Section we present the results of magneto-absorption measurements in Cu_2_O in static magnetic fields up to 32 T. In the next section we discuss crystal symmetry and the band structure of Cu_2_O. We determine the symmetry of electron and hole wave functions, which allows us to derive the optical selection rules (see Section on Selection Rules). Next, we calculate the peak positions in the absorption spectrum. The comparison between the experimental and theoretical results is discussed in the final section.

## Results

### Experimental results

The magneto-absorption of Cu_2_O has been studied in a Faraday geometry (${\bf{H}}\parallel {\bf{k}}$) with magnetic fields up to *B* = 32 T at a temperature of *T* = 1.2 K. For the experiments, platelets (thickness 40 *μ*m) cut and polished from floating zone grown Cu_2_O single crystals^23^ of [100] orientation were placed in a pumped liquid helium bath cryostat. A halogen lamp was used as a light source. Circular polarization was achieved by a combination of a quarter-wavelength plate and a polarizer situated inside the cryostat. The polarized light then was detected with a double monochromator (resolution 0.02 nm) equipped with a LN_2_ cooled CCD camera. Right (*σ*^+^) and left (*σ*^−^) circular polarization of transmitted light was resolved by switching the magnetic field direction: (${\bf{H}}\,\uparrow \,\uparrow \,{\bf{k}}$) for *σ*^+^ polarization detection and (${\bf{H}}\,\uparrow \,\downarrow \,{\bf{k}}$) for *σ*^−^ polarization detection.

**Figure 1:**
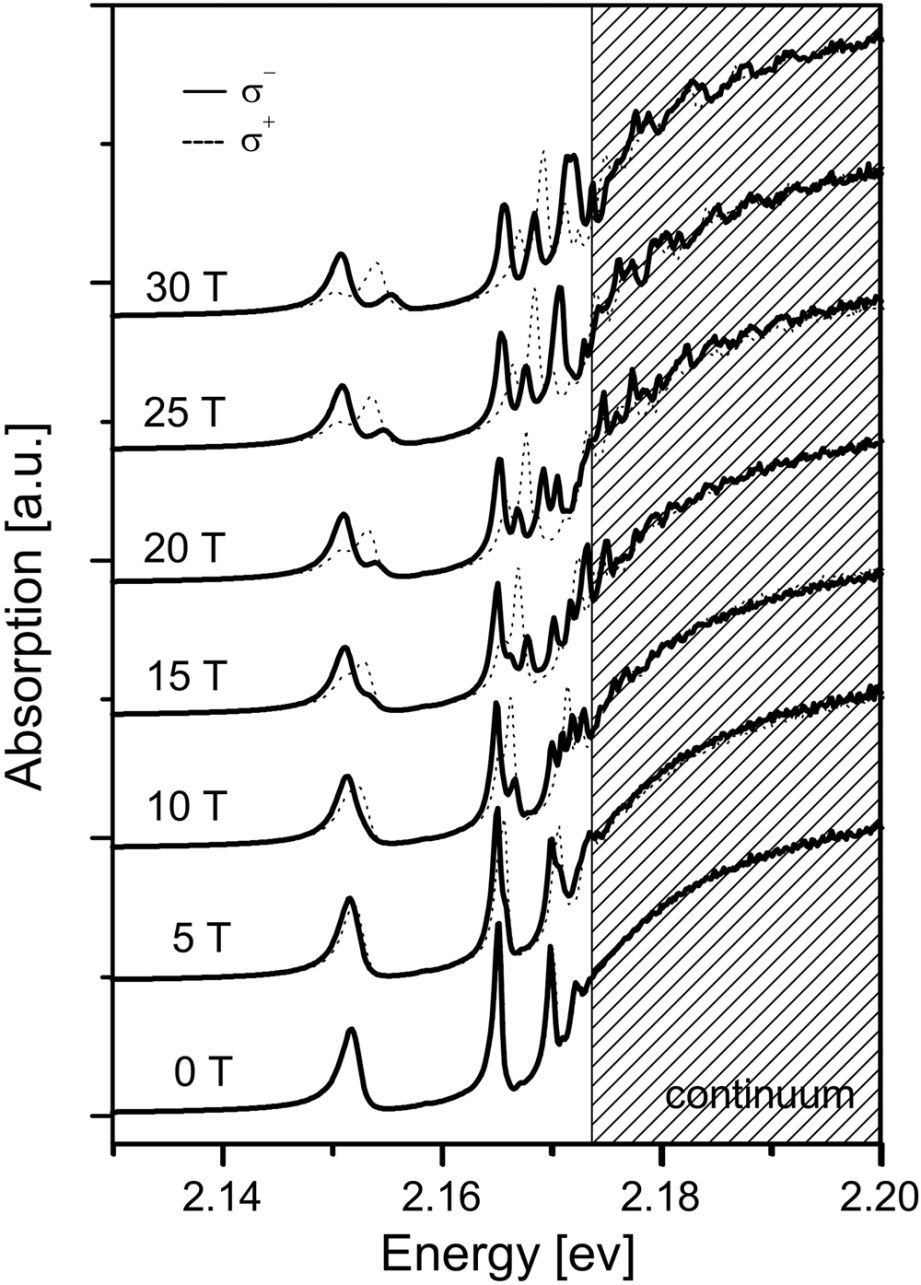
Magneto-absorption spectra of Cu_2_O in yellow exciton energy range for different magnetic field strengths. Solid line - left circular polarized spectra; dotted line - right circular polarized spectra. Bath temperature *T* = 1.2 K. The spectra for different fields have been given an offset for clarity.

Figure 1 shows the magnetic field dependence of the absorption spectra for some selected field strengths. In the absence of a magnetic field, the absorption spectrum of Cu_2_O exhibits the well known hydrogen-like absorption series below the interband transition energy. The spectral resolution of the experiments and the quality of the sample allows the observation of at least 5 exciton peaks of the yellow series (*n* = 2–6). Upon applying a magnetic field, the spectra become increasingly more complex; The exciton absorption peaks show a progressive splitting and the continuum above the band gap energy shows a complex magneto-oscillatory spectrum originating from Landau quantization of the unbound electron and hole states.

**Figure 2:**
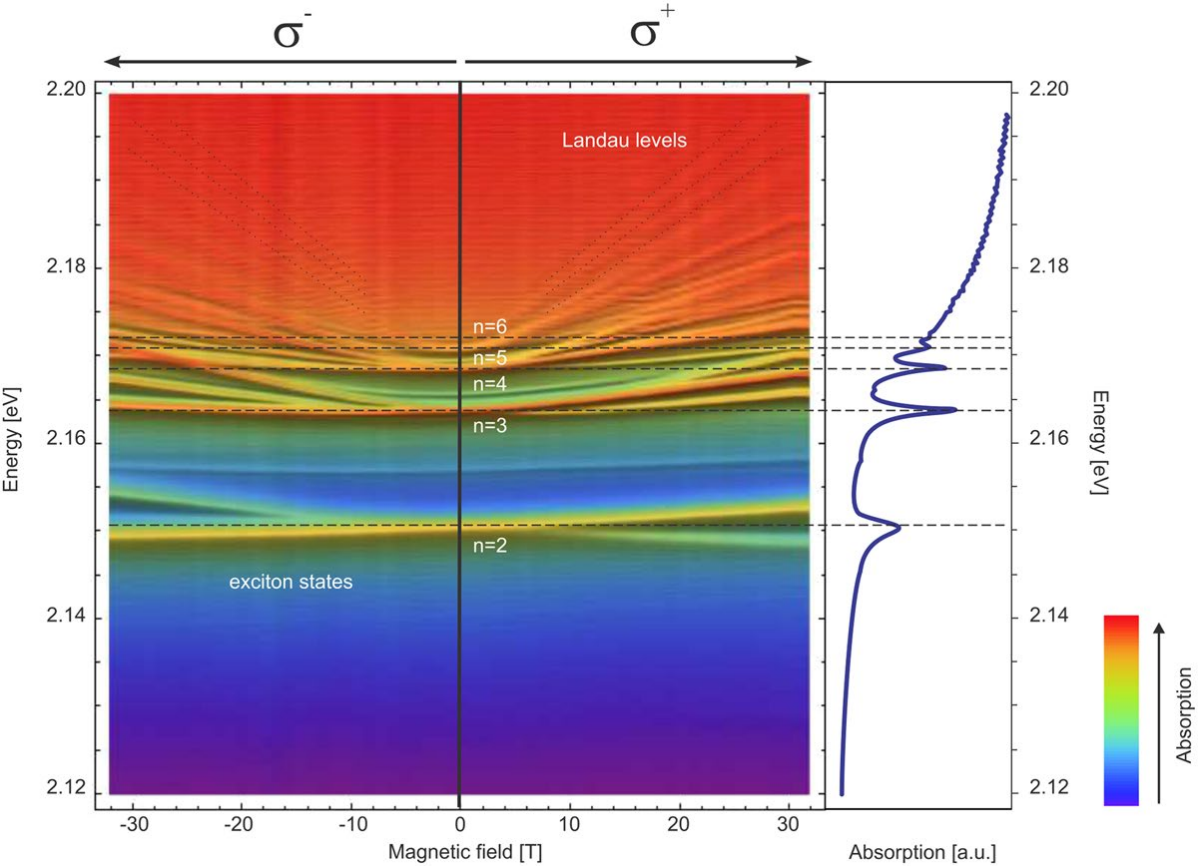
Image plot of the absorption spectra of Cu_2_O at *T* = 1.2 K. Left part - *σ*^−^ polarization; right part - *σ*^+^ polarization. The red parts are showing the higher absorption areas. The dashed lines in Landau levels region are the guides for an eye.

In order to address the complexity of the spectra, detailed measurements of the field-dependent circular polarized spectra up to B = 32 T were performed. Figure 2 represents an overview of the optical absorption spectra in a false color representation of the intensity as a function of the photon energy and magnetic field. The left part represents *σ*^−^ spectra, whereas the right part represents *σ*^+^ spectra. For the *n* = 2 state, the absorption peak shows a splitting continuous combined Zeeman and Langevin shift upon increasing magnetic field throughout the whole magnetic field range.

The absorption spectrum becomes more complex with the increase of the principal quantum number *n*. In case of Cu_2_O, only transitions to p-states for each principal quantum number *n* are dipole-allowed. These states are clearly observed in the absence of a magnetic field. With increasing magnetic field, one can observe other *l*-index (orbital number) states, due to the finite off-diagonal elements induced by the magnetic field^15^. The general behavior of the absorption peaks in this area are described in^16^. However, the diamagnetic coefficients of these peaks cannot be explained using the simple calculation based on first-order perturbation theory^16^. For *n* = 3 the situation is still relatively simple in that only three lines are observed which do not show any additional splitting upon increasing field strength. For larger *n* the exciton peaks cross or show avoided crossing behavior leading to deviations of the expected diamagnetic and Zeeman shifts^11,14–16^. Furthermore, additional absorption lines appear at high magnetic fields which will be discussed later.

The energy region in the vicinity of and above the band-gap energy is of particular interest (Fig. 2): already at 8 T equidistant quasi-Landau levels become visible. Hammura *et al*.^18^ suggested, that the electron-hole pair undergoes a periodic orbit mainly determined by the Coulomb potential which is perturbed by the presence of the magnetic field. Seyama *et al*.^16^ considered these levels as a result of frequent level crossing of states with different quantum numbers *n* and *l*, since the level spacing is comparable with the anti-crossing gaps. As described in the remainder of this paper, the proper description of the complex magnetoabsorption spectrum Fig. 2 follows directly from the gradual transition from exciton to the magneto-exciton behavior without the need for the orbital interference effects as described in^18^.

### Symmetry of the yellow excitons

**Figure 3:**
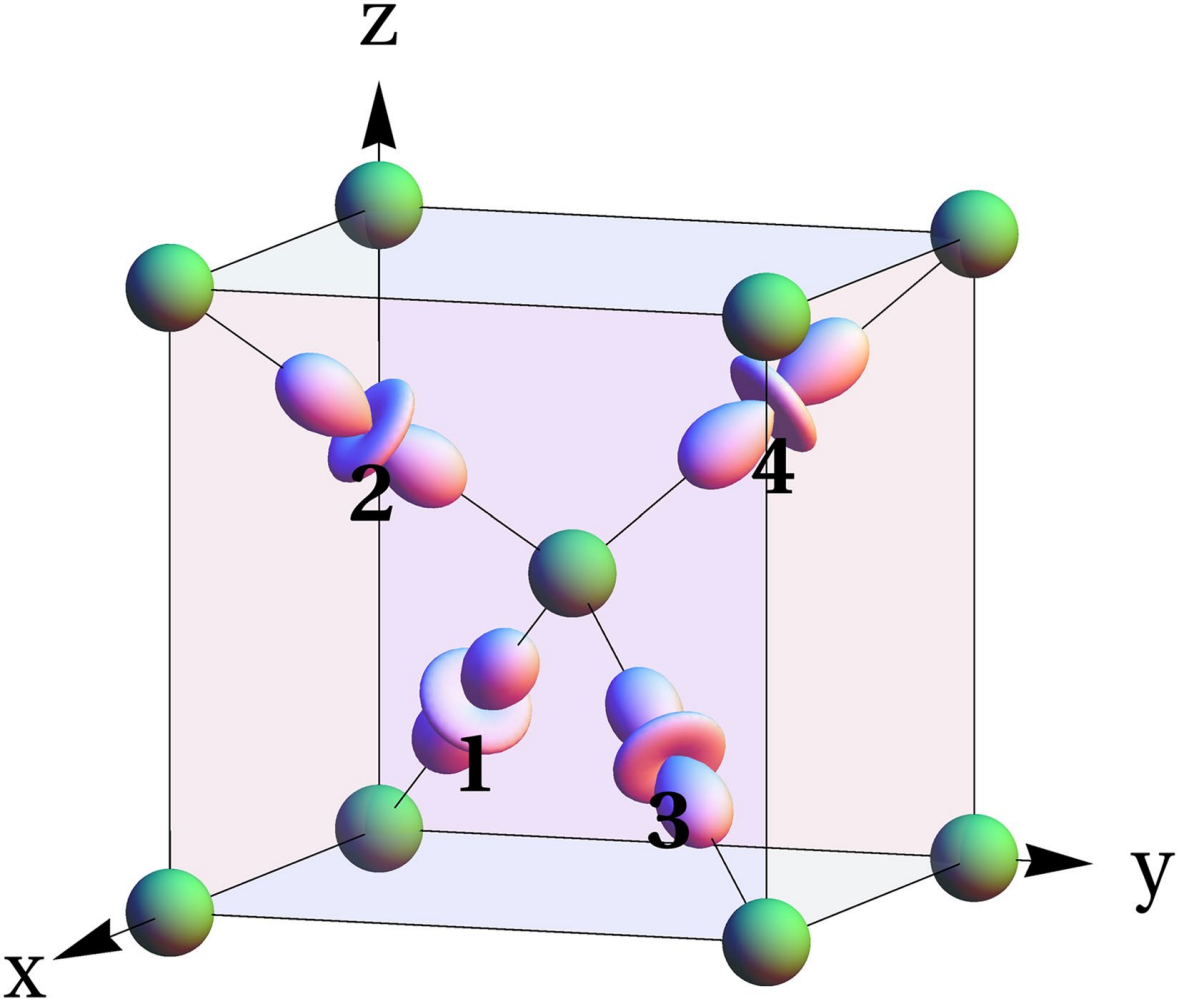
The unit cell of Cu_2_O. Oxygen ions in green form a body-centered cubic lattice. The Cu *d* orbitals (numbered) give the dominant contribution to the upper valence band.

The cuprite Cu_2_O has a cubic symmetry (space group *Pn*$\bar{3}$*m*) with four Cu ions in the unit cell (see Fig. 3). The electron can be excited from the highest valence band, formed mostly by the Cu 3*d* orbitals, to the lowest conduction band, formed by the Cu 4*s* orbitals. The yellow excitons are then formed by binding the excited electrons and holes with Coulomb interaction.

The symmetries of the electron and hole bands, respectively, ${{\rm{\Gamma }}}_{6}^{+}$ and ${{\rm{\Gamma }}}_{7}^{+}$^2^, can be understood as follows. Each Cu^+^ ion is coordinated by two oxygen ions in the dumbbell configuration, which splits its *d*-shell into two doublets, $({x}_{i}^{2}-{y}_{i}^{2},{x}_{i}{y}_{i})$ and (*x*_*i*_*z*_*i*_, *y*_*i*_*z*_*i*_), and one singlet, $3{z}_{i}^{2}-{r}_{i}^{2}$. Here the direction of the *z*_*i*_ axis is parallel to the O-Cu-O line passing through the *i*-th Cu ion (*i* = 1, 2, 3, 4) in the unit cell and is different for different Cu sites (see Fig. 3). Since the $3{z}_{i}^{2}-{r}_{i}^{2}$ state has the highest energy, we assume for simplicity that the upper valence band is formed by these orbitals only.

As the hopping amplitudes between all pairs of the $3{z}_{i}^{2}-{r}_{i}^{2}$ orbitals on neighboring Cu sites are equal by symmetry, the tight-binding band structure at the Γ-point consists of the non-degenerate singlet state,

1 $$|S\rangle =\frac{1}{2}(\mathrm{|1}\rangle +\mathrm{|2}\rangle +\mathrm{|3}\rangle +\mathrm{|4}\rangle )$$

and the triplet of degenerate states,

2 $$\{\begin{array}{rcl}|X\rangle  & = & \frac{1}{2}(\,+\,\mathrm{|1}\rangle -\mathrm{|2}\rangle -\mathrm{|3}\rangle +\mathrm{|4}\rangle ),\\ |Y\rangle  & = & \frac{1}{2}(\,-\,\mathrm{|1}\rangle +\mathrm{|2}\rangle -\mathrm{|3}\rangle +\mathrm{|4}\rangle ),\\ |Z\rangle  & = & \frac{1}{2}(\,-\,\mathrm{|1}\rangle -\mathrm{|2}\rangle +\mathrm{|3}\rangle +\mathrm{|4}\rangle ),\end{array}$$

where |*i*〉 denotes the $3{z}_{i}^{2}-{r}_{i}^{2}$ orbital on the *i*-th Cu site. Table 2 shows the transformation of these three states under the generators of $Pn\bar{3}m$ group: the *π*-rotation around the *z*-axis, *C*_2*z*_: $(x,y,z)\to (\frac{1}{2}-x,\frac{1}{2}-y,z)$, the $\frac{2\pi }{3}$-rotation around the body diagonal of the cube, *C*_3_: (*x*, *y*, *z*) → (*z*, *x*, *y*), the mirror, *m*_*x*−*y*_: (*x*, *y*, *z*) → (*y*, *x*, *z*), and inversion *I*: (*x*, *y*, *z*) → (1/2 − *x*, 1/2 − *y*, 1/2 − *z*).

**Table 2:** The transformation rules of the |*X*〉, |*Y*〉, |*Z*〉 states under the operations of $Pn\bar{3}m$ group.

State	*C*_2*z*_	*C*_3_	*m*_*x*−*y*_	*I*
|*X*〉	−|*X*〉	|*Y*〉	|*Y*〉	|*X*〉
|*Y*〉	−|*Y*〉	|*Z*〉	|*X*〉	|*Y*〉
|*Z*〉	+|*Z*〉	|*X*〉	|*Z*〉	|*Z*〉

As the hopping between nearest-neighbor Cu sites is mediated by oxygen ions, the hopping parameter *t* of the effective tight-binding model describing the Cu sites only,

$$H=-\,t\,\sum _{\langle i,j\rangle \sigma }\,({c}_{i\sigma }^{\dagger }{c}_{j\sigma }+{c}_{j\sigma }^{\dagger }{c}_{i\sigma }),$$

where the operator *c*_*iσ*_ annihilates electron in the state $3{z}_{i}^{2}-{r}_{i}^{2}$ on the site *i* with the spin projection *σ*, is given by $t=\frac{{t}_{pd}^{2}}{{\rm{\Delta }}}$, where *t*_*pd*_ is the hopping amplitude between the Cu to O sites and Δ > 0 is the charge transfer energy. Since *t* > 0, the states with the energy +2*t* at the Γ-point lie higher than the singlet state with the energy −6*t*. The spin-orbit interaction further splits the six (including the spin degeneracy) states into a doublet^3^,

3 $$\{\begin{array}{rcl}{|\uparrow \rangle }_{v} & = & -\frac{1}{\sqrt{3}}[(|X\rangle +i|Y\rangle )|\downarrow \rangle +|Z\rangle |\uparrow \rangle ],\\ {|\downarrow \rangle }_{v} & = & \frac{1}{\sqrt{3}}[(\,-\,|X\rangle +i|Y\rangle )|\uparrow \rangle +|Z\rangle |\downarrow \rangle ],\end{array}$$

and a quadruplet (here the subscript *v* indicates the valence band). The energy of the doublet is higher than the energy of the quadruplet by ~134 meV^24^. This spin-orbital splitting originates from the virtual admixture of *x*_*i*_*z*_*i*_ and *y*_*i*_*z*_*i*_ states to the $3{z}_{i}^{2}-{r}_{i}^{2}$ state by the spin-orbit coupling on Cu sites. The doublet belongs to the upper valence band, which gives rise to the yellow exciton, while the quadruplet gives rise to the green exciton series^17^. We stress that our |*X*〉, |*Y*〉 and |*Z*〉 states are formed by the $3{z}_{i}^{2}-{r}_{i}^{2}$ orbitals of the four Cu ions in the unit cell and are different from the atomic *xy*, *yz*, and *zx* orbitals discussed by Kavoulakis *et al*.^3^.

Using Eq. (3) and Table 2, one finds that the valence-band doublet, $${\psi }_{v}=(\begin{array}{c}|\,\uparrow \,{\rangle }_{v}\\ |\,\downarrow \,{\rangle }_{v}\end{array})$$, transforms as a ${{\rm{\Gamma }}}_{7}^{+}$ representation:

4 $$\begin{array}{rcl}{C}_{2z}{\psi }_{v} & = & {e}^{-i\frac{\pi }{2}{\sigma }_{z}}{\psi }_{v}=(\begin{array}{cc}-i & 0\\ 0 & i\end{array})\,{\psi }_{v},\\ {C}_{3}{\psi }_{v} & = & {e}^{-i\frac{\pi }{3\sqrt{3}}({\sigma }_{x}+{\sigma }_{y}+{\sigma }_{z})}\,{\psi }_{v}=\frac{1}{2}\,(\begin{array}{cc}1-i & -1-i\\ 1-i & 1+i\end{array})\,{\psi }_{v},\\ {m}_{x-y}{\psi }_{v} & = & \frac{i}{\sqrt{2}}({\sigma }_{x}-{\sigma }_{y})\,{\psi }_{v}=\frac{i}{\sqrt{2}}\,(\begin{array}{cc}0 & 1+i\\ 1-i & 0\end{array})\,{\psi }_{v},\\ I{\psi }_{v} & = & {\psi }_{v}\mathrm{.}\end{array}$$

Similarly, the lowest conduction band, formed by the Cu 4*s* orbitals, splits into a triplet and singlet at the Γ-point with the singlet state having a lower energy. Since the orbital part of the singlet wave function [see Eq. (1)], is invariant under all operations of the space group, the symmetry of the doublet, $${\psi }_{c}=(\begin{array}{c}|\,\uparrow \,{\rangle }_{c}\\ |\,\downarrow \,{\rangle }_{c}\end{array})$$, formed by the spin-up and spin-down electron states in the lowest conduction band, is determined by its spin wave function. Thus, the conduction electron in the yellow exciton has the same transformation properties as the valence electron [see Eq. (4)], except for the opposite sign for the mirror transformation, *m*_*x*−*y*_, and, hence, belongs to ${{\rm{\Gamma }}}_{6}^{+}$ representation.

Finally, the conduction electron and the valence hole form ortho- and para-excitons with the total spin, *S*, respectively, 1 and 0. Due to the exchange interaction between the conduction and valence electrons in the *n* = 1 yellow exciton state, the energy of the ortho-exciton is 12 meV higher than that of para-exciton^3,25–28^.

### Selection rules

Since the valence 3*d* and conduction 4*s* bands have the same parity, the excitation of the yellow exciton series is dipole forbidden and results from the electric quadrupole transition^2^. The conduction and valence band doublets, *ψ*_*c*_ and *ψ*_*v*_, transform under the mirror *m*_*x*−*y*_ with opposite signs (see the Symmetry Section), resulting in the“wrong” symmetry of yellow excitons: the paraexciton wave function,

$$|S=0,{S}_{z}=0\rangle =\frac{1}{\sqrt{2}}\,({\psi }_{c\uparrow }{\psi }_{v\downarrow }-{\psi }_{c\downarrow }{\psi }_{v\uparrow }),$$

is odd under *m*_*x*−*y*_, while the orthoexciton wave function with zero projection of the total spin,

$$|S=1,{S}_{z}=0\rangle =\frac{1}{\sqrt{2}}({\psi }_{c\uparrow }{\psi }_{v\downarrow }+{\psi }_{c\downarrow }{\psi }_{v\uparrow }),$$

is even.

The invariance of the paraexciton wave function |0, 0〉 under *C*_3_ and *C*_2_ rotations requires that the amplitude of the photoexcitation of this state has the form,

5 $${A}_{00}\propto \sum _{{\bf{k}}}\,{{\boldsymbol{\phi }}}_{{\bf{k}}}^{\ast }({\bf{e}}\cdot {\bf{k}}),$$

where **k** is the relative wave vector of the electron-hole pair, ***φ***_**k**_ is the wave function of the relative motion, discussed in the next section, and **e** = **e**_**q***λ*_ is the polarization vector of the photon with the wave vector **q** and polarization *λ*. The scalar product (**e** · **k**) is invariant under *m*_*x*−*y*_, while *A*_00_ must be odd, implying that *A*_00_ = 0, i.e., paraexcitons cannot be excited via the one-photon absorption.

The orthoexciton states |1, *S*_*z*_〉 with *S*_*z*_ = −1, 0, 1, are excited by the components of the quadrupolar tensor,

6 $${Q}_{ab}\propto \sum _{{\bf{k}}}\,{{\boldsymbol{\phi }}}_{{\bf{k}}}^{\ast }\,({e}_{a}{k}_{b}+{e}_{b}{k}_{a}-\frac{2}{3}{{\boldsymbol{\delta }}}_{ab}{\bf{e}}\cdot {\bf{k}}).$$

The excitation amplitudes, invariant under all crystal symmetries, have an obvious form for the Cartesian components of the orthoexciton atomic wave functions, |x〉, |y〉, and |z〉:

7 $$\{\begin{array}{lcl}|1,1\rangle  & = & -\frac{1}{\sqrt{2}}(|{\rm{x}}\rangle +i|{\rm{y}}\rangle ),\\ |1,0\rangle  & = & |{\rm{z}}\rangle ,\\ |1,-\,1\rangle  & = & \frac{1}{\sqrt{2}}(|{\rm{x}}\rangle -i|{\rm{y}}\rangle ).\end{array}$$

The form of the invariant amplitudes is:

8 $$\begin{array}{lll}{A}_{{\rm{x}}} & \propto  & {Q}_{yz},\\ {A}_{{\rm{y}}} & \propto  & {Q}_{zx},\\ {A}_{{\rm{z}}} & \propto  & {Q}_{xy},\end{array}$$

and the proportionality coefficient is the same for all states.

For the Faraday geometry, $q\parallel H$ (and $H\parallel z$),

9 $${A}_{x},{A}_{y}\propto \sum _{{\bf{k}}}\,{k}_{z}{{\boldsymbol{\phi }}}_{{\bf{k}}}^{\ast }\propto {\frac{\partial {{\boldsymbol{\phi }}}^{\ast }}{\partial z}|}_{{\bf{r}}=0},$$

so that these amplitudes are only nonzero for *m* = 0, where *m* is the *z*-projection of orbital momentum of the relative motion of the electron-hole pair. Similarly, *A*_*z*_, does not vanish only for *m* = ±1 states with nonzero ${[\frac{\partial {\phi }^{\ast }}{\partial x}\mp i\frac{\partial {\phi }^{\ast }}{\partial y}]}_{{\bf{r}}=0}$. For zero magnetic field, the allowed excited states have the orbital momentum *l* = 1 (*p*-states).

In this way we can obtain the following unusual selection rules for orthoexcitons from the yellow series: a photon with the polarization *λ* = ±1 $[{{\bf{e}}}_{{\bf{q}},\pm 1}=\frac{1}{\sqrt{2}}(1,\pm \,i,0)]$ excites either the state with *S*_*z*_ = −*λ* and *m* = 0, or the state with *S*_*z*_ = 0 and *m* = −*λ*. These selection rules are opposite to those for rotationally-invariant systems, where the *z*-component of the total angular momentum is a good quantum number.

### Motion of electron-hole pair in magnetic field

The atomic part of the exciton wave function, discussed in the previous section, remains largely unaffected by an applied magnetic field of 32 T, except for the mixing of the paraexciton and orthoexciton states. On the other hand, magnetic field has a strong effect on the relative motion of the electron and hole, especially in highly-excited excitonic states. The problem of finding energies of excitonic states in magnetic field is simplified by the conservation of the total momentum of the electron-hole pair^29^, which makes it equivalent to the problem of a hydrogen atom in a magnetic field^30,31^.

The relatively slow motion of electron and hole in the Cu_2_O excitonic states is, to a good approximation, decoupled from the dynamics of their spins and can be considered separately. The Lagrangian describing this motion is

10 $$\begin{array}{rcl}L & = & \frac{{m}_{e}{\dot{{\bf{r}}}}_{e}^{2}}{2}+\frac{{m}_{h}{\dot{{\bf{r}}}}_{h}^{2}}{2}-\frac{e}{c}{\bf{A}}({{\bf{r}}}_{e})\cdot {\dot{{\bf{r}}}}_{e}+\frac{e}{c}{\bf{A}}({{\bf{r}}}_{h})\cdot {\dot{{\bf{r}}}}_{h}\\  &  & +\frac{{e}^{2}}{\varepsilon |{{\bf{r}}}_{e}-{{\bf{r}}}_{h}|},\end{array}$$

where **r**_*e*_(**r**_*h*_) is the electron(hole) coordinate, *m*_*e*_ and *m*_*h*_ are the electron and hole masses, and ${\bf{A}}({\bf{r}})=\frac{1}{2}[{\bf{H}}\times {\bf{r}}]$ is the vector potential (the electron charge is −*e*).

In the center-of-mass and relative coordinates, ${\bf{R}}=\frac{{{\bf{r}}}_{e}{m}_{e}+{{\bf{r}}}_{h}{m}_{h}}{{m}_{e}+{m}_{h}}$ and **r** = **r**_*e*_ − **r**_*h*_, the Lagrangian has the form,

11 $$L=\frac{M{\dot{{\bf{R}}}}^{2}}{2}+\frac{\mu {\dot{{\bf{r}}}}^{2}}{2}-\frac{e}{c}(\dot{{\bf{R}}}+\frac{\gamma }{2}\dot{{\bf{r}}})\cdot [{\bf{H}}\times {\bf{r}}]+\frac{{e}^{2}}{\varepsilon r},$$

where *M* = *m*_*e*_ + *m*_*h*_ and $\mu =\frac{{m}_{e}{m}_{h}}{{m}_{e}+{m}_{h}}$ are, respectively, the total and the reduced mass of the electron-hole pair,

12 $$\gamma =\frac{{m}_{h}-{m}_{e}}{{m}_{h}+{m}_{e}},$$

and the total time derivative $\frac{e}{2c}\frac{d}{dt}({\bf{r}}\cdot [{\bf{H}}\times {\bf{R}}])$ was omitted from the Lagrangian.

The corresponding Hamiltonian is

13 $$H=\frac{1}{2M}{({\bf{P}}+\frac{e}{c}[{\bf{H}}\times {\bf{r}}])}^{2}+\frac{1}{2\mu }{({\bf{p}}+\frac{e\gamma }{2c}[{\bf{H}}\times {\bf{r}}])}^{2}-\frac{{e}^{2}}{\varepsilon r},$$

where ${\bf{P}}=M\dot{{\bf{R}}}-\frac{e}{c}[{\bf{H}}\times {\bf{r}}]$ and ${\bf{p}}=\mu \dot{{\bf{r}}}-\frac{e\gamma }{2c}[{\bf{H}}\times {\bf{r}}]$ are, respectively, the total and relative momenta. The Hamiltonian is independent of the center-of-mass coordinate **R**, which makes the total momentum **P** an integral of motion. Since only the excitons with **P** = 0 are directly excited in an optical experiment, the Hamiltonian can be written in the form,

14 $$H=\frac{{{\bf{p}}}^{2}}{2\mu }+\frac{e}{2\mu c}{\bf{L}}\cdot (\gamma {\bf{H}})-\frac{{e}^{2}}{\varepsilon r}+\frac{{e}^{2}}{8\mu {c}^{2}}{[{\bf{H}}\times {\bf{r}}]}^{2},$$

where **L** = [**r** × **p**] is the orbital momentum. Equation (14) has the form of the Hamiltonian of an electron in the hydrogen atom in a magnetic field *γ***H** and in a parabolic trapping potential in the plane perpendicular to **H** (the last term in Eq. (14) also known as the Langevin or diamagnetic term).

For convenience we choose the cylindrical coordinates with the *z* axis along the magnetic field, and $\rho =\sqrt{{x}^{2}+{y}^{2}}$. The Hamiltonian (14) is invariant under rotations around the direction of magnetic field, therefore $m=\frac{1}{\hslash }{L}_{z}$ is a good quantum number. As was discussed in Selection Rules Section, only the exciton states with *m* = 0, ±1 are excited in the photoabsorption experiment.

The dependence of eigenfunctions on *z* and *ρ* was found numerically by solving eigenvalue problem for the Hamiltonian written in the basis of functions,

15 $${\psi }_{m{n}_{\rho }{n}_{z}}(\rho ,z)={e}^{-\frac{({z}^{2}+{\rho }^{2})}{2{l}^{2}}}\,{(\frac{\rho }{l})}^{|m|}{L}_{{n}_{\rho }}^{|m|}\,(\frac{{\rho }^{2}}{{l}^{2}})\,{H}_{{n}_{z}}\,(\frac{z}{l}),$$

where $l=\sqrt{\frac{2\hslash c}{eH}}$ is the magnetic length ${l}_{0}=\sqrt{\frac{\hslash c}{eH}}$ multiplied by $\sqrt{2}$, while ${H}_{{n}_{z}}$ and ${L}_{{n}_{\rho }}^{|m|}$ are, respectively, the Hermite and Laguerre polynomials. In this basis the matrix elements of the Coulomb interaction can be evaluated analytically, which simplifies the calculation of the eigenstates of the Hamiltonian (14).

This method is appropriate in the strong-field limit, where the distance between the Landau levels, $\frac{\hslash eH}{\mu c}$, is larger than the exciton Rydberg constant, ${{\rm{Ry}}}_{{\rm{X}}}=\frac{\mu {e}^{4}}{2{\varepsilon }^{2}{\hslash }^{2}}$. However, using a rather large basis with *n*_*ρ*_ ≤ 10 and *n*_*z*_ ≤ 10, we can extend its applicability up to the physically interesting fields of ~15 T. In the opposite limit of weak fields, we diagonalize the Hamiltonian (14) in the basis of the zero-field hydrogen wave functions of the discrete spectrum and truncate the basis at *n* = 20. In both cases we checked that the energies of the levels do not change upon a further increase of the basis dimension. The *H*-dependence of the exciton energies obtained in the two opposite limits matches in the region of intermediate magnetic fields, which allows us to calculate the exciton energies for arbitrary magnetic fields. The dashed lines in Fig. 5 show the magnetic field dependence of the excitonic levels calculated by numerical diagonalization of the Hamiltonian (14) superimposed on experimental absorption spectra. The red dots indicate the points of a crossover between high- and low-field lines.

### Fit to experimental data

In order to fit the experimental data, it is necessary to take into account the field dependence of the excitonic energies resulting from the interaction of the electron and hole spins with the magnetic field $H\parallel z$:

16 $${\hat{H}}_{{\rm{spin}}}={\mu }_{{\rm{B}}}H({g}_{c}{j}_{e}^{z}+{g}_{v}{j}_{v}^{z}),$$

where ${j}_{c}^{z}$ and ${j}_{v}^{z}$ are the *z*-components of the angular momenta of the conduction and valence electron forming the exciton and *g*_*c*_ and *g*_*v*_ are respectively the *g*-factors of electrons in the conduction and valence band. The interaction of spins with the magnetic field mixes the ortho |1, 0〉 and para |0, 0〉 states and the corresponding energies are:

$${E}_{\pm }={E}_{0}\pm \sqrt{{(\frac{{{\rm{\Delta }}}_{{\rm{o}}-{\rm{p}}}}{2})}^{2}+{[\frac{1}{2}({g}_{c}-{g}_{v}){\mu }_{{\rm{B}}}B]}^{2}}$$

where Δ_o−p_ is the exchange splitting between the ortho and para states in zero field. It is proportional to the square of the enveloping electron-hole wavefunction *φ* at *r* = 0^3^, which is only nonzero for *s*-states, whereas electric quadrupole excitation is only allowed to *p*-states [see Eq. (9)]. In fact, existing experimental data on the yellow exciton series shows that the ortho-para splitting is zero for *n* > 1 within the experimental precision^32^.

Therefore, in an applied magnetic field the spin part of the exciton wave functions has the form,

17 $$\begin{array}{rcl}{\psi }_{+} & = & \frac{|10\rangle +|00\rangle }{\sqrt{2}}=|{\uparrow }_{c}\rangle |{\downarrow }_{v}\rangle ,\\ {\psi }_{-} & = & \frac{|10\rangle -|00\rangle }{\sqrt{2}}=|{\downarrow }_{c}\rangle |{\uparrow }_{v}\rangle ,\end{array}$$

which allows us to extract the *g*-factors of electrons in the conduction and valence band (see Discussion Section).

Furthermore, to extract the exciton parameters it is important to take into account that the coupling of excitons to the lattice modifies the shape of the absorption peaks, and shifts the maximum of the absorption away from the position in the rigid lattice. The lineshape can be fitted with the asymmetric Lorenzian^33^,

18 $$I(\omega )\sim \frac{\hslash {\rm{\Gamma }}/2+2A(\hslash \omega -E)}{{(\hslash \omega -E)}^{2}+{(\hslash {\rm{\Gamma }}/2)}^{2}},$$

where *E* is the exciton energy in the “rigid” lattice, Γ is the exciton-phonon scattering strength and *A* is the asymmetry parameter.

**Figure 4:**
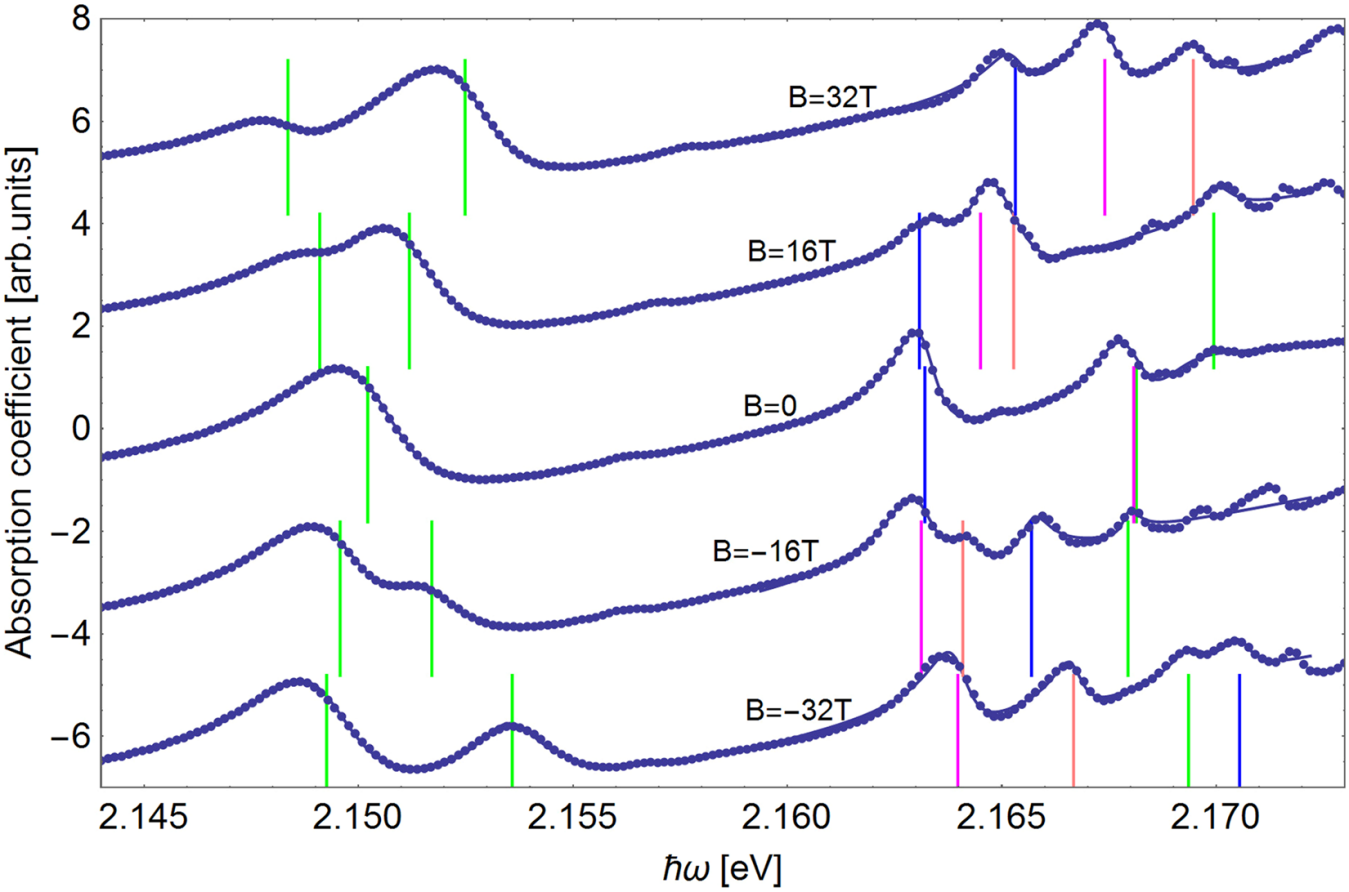
The absorption spectrum for the *n* = 2, 3 exciton (dots) at various values of magnetic field fitted with the sum of asymmetric Lorenzian peaks (solid line) The ‘bare’ exciton energies (see text) are indicated by vertical lines. The fit quality parameter is *R*^2^ > 0.999997 for the n = 2 levels and *R*^2^ > 0.9996 for *n* = 3 levels.

The fit of the absorption spectrum for the *n* = 2, 3 excitons at various values of magnetic field is shown in Fig. 4, where circles represent the experimental data and the continuous line is a fit by Eq. (18). The linewidth Γ = 2 meV for *n* = 2 levels agrees with the results of earlier studies^34^.

The excellent quality of the fit allows us to extract the ‘bare’ exciton energies, indicated by vertical lines. Since the maxima of the absorption spectra are displaced with respect to the bare exciton energies, this procedure enables us to extract the *g*-factors and masses of the electron and hole from the experimental data in a more reliable way.

## Discussion

**Figure 5:**
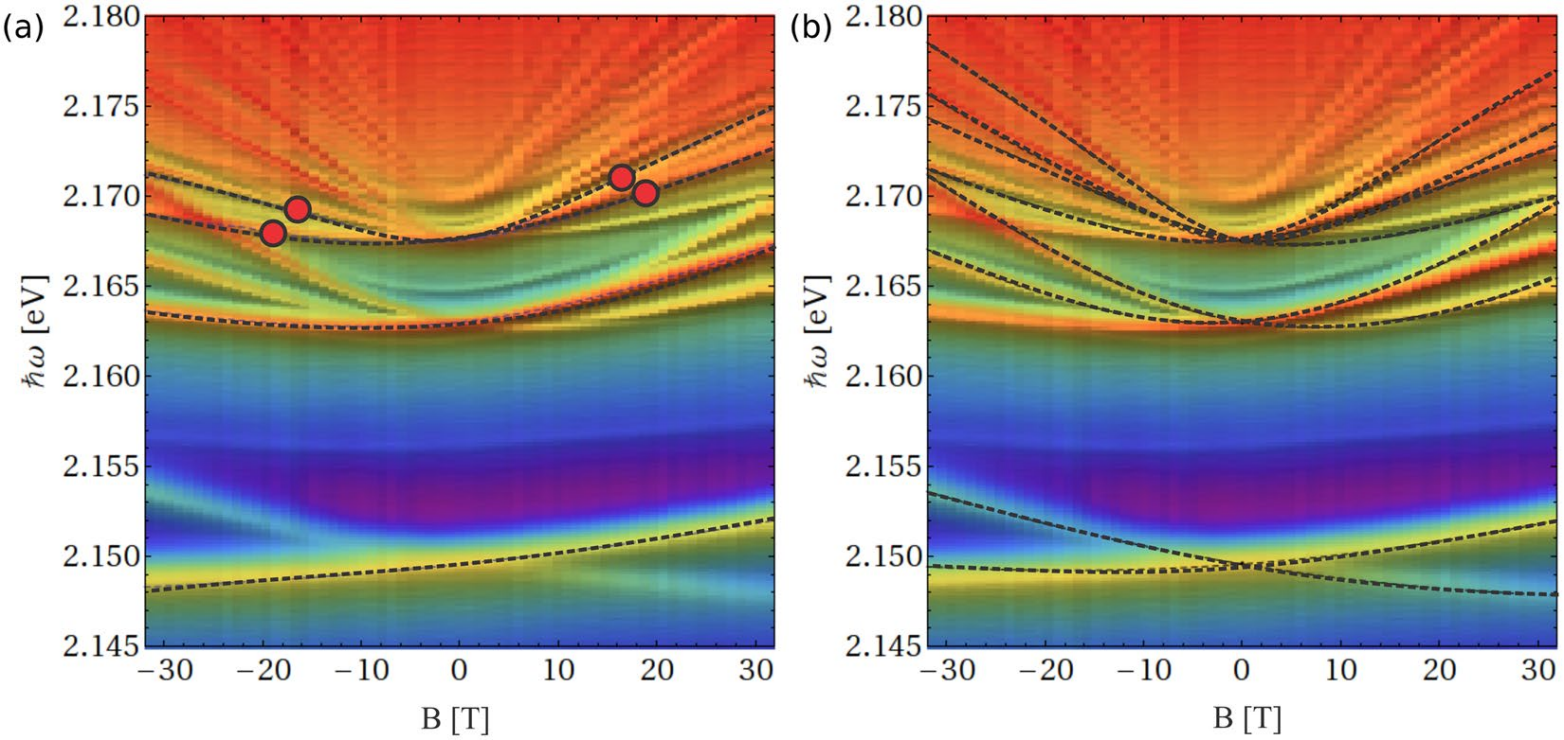
The magnetoabsorption spectra measured in the Faraday geometry, ${\bf{H}}\parallel {\bf{q}}\parallel (001)$, together with the theoretically calculated magnetic field dependence of the energies (dashed lines) for the excitons with [panel (a), set 1] *S*_*z*_ = −1 and *m* = 0 and [panel (b), set 2] *S*_*z*_ = 0 and *m* = −1. Red disks represent the points where strong and weak magnetic field solutions match.

Figure 5 shows the magnetic field and photon energy dependence of the optical absorption with the calculated excitonic energies superimposed. In accordance with the selection rules, *n* ≥ 2 excitons contribute to the optical absorption, forming at zero magnetic field a hydrogen-like series $\hslash {\omega }_{n}={E}_{gap}-\frac{{\rm{R}}{y}_{X}}{{n}^{2}}$ with the optical band gap *E*_*gap*_ = 2.172 eV and the excitonic Rydberg constant Ry_X_ = 98 meV. [The binding energy of *n* = 1 exciton is anomalously large (150 meV). The exciton radius of the *n* = 1 exciton (7 Å) is comparable to the lattice constant (4.2 Å), which leads to significant central cell corrections and reduced screening of Coulomb interaction responsible for this anomaly^3^. The corrections to the binding energy of the *n* = 2 level, produced by these mechanisms, are negligible]. Using *ε* = 7.5 for the dielectric constant^11,13,28^, we obtain the reduced mass *μ* = 0.41*m*_0_ in agreement with ref.^12^.

According to the selection rules derived in the Section on Selection Rules, the absorption spectrum for the right circularly-polarized light (*λ* = +1) is formed by two different sets of states: the states with *S*_*z*_ = −1 and *m* = 0 (set 1) and the states with *S*_*z*_ = 0 and *m* = +1 (set 2). Dashed lines in Fig. 5 show the numerically calculated energies of *n* = 2, 3, 4 exciton states in magnetic field up to 32 T, which belong, respectively, to the sets 1 and 2, superimposed on the experimental absorption spectra.

Set 1 corresponds to the absorption of a photon with *λ* = +1 and creation of an exciton in the state |1, −1〉, *m* = 0. The magnetic moment in this state is determined by the atomic g-factors of electrons and holes. Since the hole in the upper valence band has *s*_*z*_ = 1/2 and *l*_*z*_ = −1, it has zero g-factor since (*l*_*z*_ + 2*s*_*z*_) = 0 ^35^. The electron wave function is mostly of Cu *s* character, and since in this case the spin-orbit interaction is not effective, the g-factor should be close to the bare value of 2. Indeed, a good agreement with the experiment is obtained for *g*_*c*_ = 2.0 (see Fig. 5b).

The last term in the Hamiltonian Eq. (14) mixes the state |*l*, *m*〉 with the states |*l*, *m*〉 and |*l* + 2, *m*〉. This leads to the mixing of *p* and *f* states for *n* ≥ 4 giving rise to additional lines. In general the line with the main quantum number *n* splits in a magnetic field into $[\tfrac{n}{2}]$ levels (here [*x*] denotes the largest integer smaller than *x*).

Set 2 of the absorption lines is produced by the |1, 0〉 ± |0, 0〉, *m* = 1 excitonic transitions. This set has twice as many states, corresponding to $|\,{\uparrow }_{c}\,{\downarrow }_{v}\,\rangle$ and $|\,{\downarrow }_{c}\,{\uparrow }_{v}\,\rangle$. The energy shifts of these levels up to the terms linear in the magnetic field are

$$E=2\,(\frac{{m}_{0}}{{m}_{h}}-\frac{{m}_{0}}{{m}_{e}})\,{\mu }_{B}Hm\pm \frac{1}{2}|{g}_{c}-{g}_{v}|{\mu }_{B}H.$$

We extracted *m*_*e*_ = 1.0*m*_0_, *m*_*h*_ = 0.7*m*_0_, |*g*_*c*_ − *g*_*v*_| = 2.25 and *g*_*c*_ + *g*_*v*_ = 2.0, so that for the atomic g-factors of electrons in the conduction and valence bands we obtain, respectively, *g*_*c*_ = 2.1, *g*_*v*_ = −0.1 [see Table 1] in good agreement with our simple arguments given above. The effective masses coincide with the results of the cyclotron resonance experiments^13^. These values of the parameters result in good agreement between the calculated and the measured spectra.

To conclude, we studied the magneto-absorption spectrum of cuprous oxide in high magnetic fields, calculated excitonic energies for arbitrary field values, and extracted the exciton parameters from the intermediate field region, where the peaks are clearly discernible. Our results suggest that the wide *n* = 2 line is a result of the overlap of two lines with different quantum numbers, resolving a controversy over the degeneracy of this level. This observation allows us to extract the masses of electrons and holes, which are consistent with the results of cyclotron resonance experiments, and g-factors consistent with the present understanding of the nature of valence and conduction bands of Cu_2_O. We hope that the presented experimental data and the level assignment serves as a benchmark for future ab-initio calculations.
